# Psychosocial job quality, mental health, and subjective wellbeing: a cross-sectional analysis of the baseline wave of the Australian Longitudinal Study on Male Health

**DOI:** 10.1186/s12889-016-3701-x

**Published:** 2016-10-31

**Authors:** Anthony D. LaMontagne, Allison Milner, Lauren Krnjacki, Marisa Schlichthorst, Anne Kavanagh, Kathryn Page, Jane Pirkis

**Affiliations:** 1 Centre for Population Health Research, School of Health and Social Development, Deakin University, Burwood, VIC 3125 Australia; 2 Centre for Health Equity, Melbourne School of Population and Global Health, The University of Melbourne, Melbourne, 3010 Australia; 3 Centre for Epidemiology and Biostatistics, Melbourne School of Population and Global Health, The University of Melbourne, Melbourne, 3010 Australia; 4 Centre for Mental Health, Melbourne School of Population and Global Health, The University of Melbourne, Melbourne, 3010 Australia

## Abstract

**Background:**

Employment status and working conditions are strong determinants of male health, and are therefore an important focus in the Australian Longitudinal Study on Male Health (Ten to Men). In this paper, we describe key work variables included in Ten to Men, and present analyses relating psychosocial job quality to mental health and subjective wellbeing at baseline.

**Methods:**

A national sample of males aged 10 to 55 years residing in private dwellings was drawn using a stratified multi-stage cluster random sample design. Data were collected between October 2013 and July 2014 for a cohort of 15,988 males, representing a response fraction of 35 %. This analysis was restricted to 18–55 year old working age participants (*n* = 13,456). Work-related measures included employment status, and, for those who were employed, a number of working conditions including an ordinal scale of psychosocial job quality (presence of low job control, high demand and complexity, high job insecurity, and low fairness of pay), and working time-related stressors such as long working hours and night shift work. Associations between psychosocial job quality and two outcome measures, mental ill-health and subjective wellbeing, were assessed using multiple linear regression.

**Results:**

The majority of participants aged 18–55 years were employed at baseline (85.6 %), with 8.4 % unemployed and looking for work, and 6.1 % not in the labour force. Among employed participants, there was a high prevalence of long working hours (49.9 % reported working more than 40 h/week) and night shift work (23.4 %). Psychosocial job quality (exposure to 0/1/2/3+ job stressors) prevalence was 36 %/ 37 %/ 20 %/ and 7 % of the working respondents. There was a dose–response relationship between psychosocial job quality and each of the two outcome measures of mental health and subjective wellbeing after adjusting for potential confounders, with higher magnitude associations between psychosocial job quality and subjective wellbeing.

**Conclusions:**

These results extend the study of psychosocial job quality to demonstrate associations with a global measure of subjective wellbeing. Ten to Men represents a valuable new resource for the longitudinal and life course study of work and health in the Australian male population.

## Background

Employment status and working conditions are powerful determinants of adult male health [1–3], and are therefore an important focus in the Australian Longitudinal Study on Male Health (Ten to Men). This paper describes key work variables and the rationale for their selection and inclusion in Ten to Men, and presents analyses comparing the relationship between psychosocial job quality and mental health, defined in terms of mental ill-health to the relationship between psychosocial job quality and subjective wellbeing which has had little attention to date.

Over the last two decades, psychosocial working conditions have emerged as leading contributors to work-related illness [4–6]. This is partly because they are universal occupational exposures that can occur in any work context, as opposed to exposures that are associated with particular occupations or work contexts (e.g., working at heights, being exposed to ionising radiation). Consequently, a substantial proportion of the working population is exposed to poor psychosocial working conditions or job stressors, including low job control, excessive job demands, job insecurity, low social support at work, and bullying [7, 8]. In addition, specific job stressors can be related to multiple health and health behavioural outcomes (e.g., low job control is predictive of cardiovascular health outcomes, depression, and anxiety) [7]. For these reasons, we have included measures of psychosocial job stressors for those in paid employment in Ten to Men. We selected a recently-developed measure which captures perceived job control, job demands and complexity, job insecurity, and fairness of pay in an index of psychosocial job quality [9, 10]; this measure has been shown to have predictive validity in relation to mental and physical health [11] as well as sickness absence [12]. Incorporating elements of the two most widely studied and validated measures of psychosocial working conditions, the demand-control model of Karasek and Theorell [13] and the effort-reward imbalance model of Siegrist [14], this measure efficiently captures multiple job stressor exposures and enables the study of the impacts of multiple job stressor co-exposures, which often occur in the working population but are understudied.

Thus far, the vast majority of research on job stressors and health has been on health problems, diseases or disorders. In contrast to this illness focus, there is growing interest in positive mental health and wellbeing outcomes in the employed population [15–19]. Wellbeing is a very broad concept, with no single consensus definition. Its contemporary historic roots include the World Health Organisation’s 1948 definition of health as a “a state of complete physical, mental, and social wellbeing and not merely the absence of disease or infirmity” [20] and Jahoda’s 1958 landmark book on positive mental health [21]; these are capsulized in WHO’s more recent definition of mental health as “a state of well-being in which the individual realizes his or her own abilities, can cope with the normal stresses of life, can work productively and fruitfully, and is able to make a contribution to his or her community” [22]. In short, these concepts attempt to complement and extend the historically dominant disease model of health, which insufficiently defines health as the absence of illness [19].

The various concepts of wellbeing (also referred to as positive mental health) include both subjective and objective dimensions and measures, with most attention being directed to subjective measures. Subjective measures include positive emotion or satisfaction dimensions (including satisfaction with life as a whole), as well as social and psychological functioning dimensions, including perceptions of meaningfulness and sense of purpose in life) [15, 16, 19, 23, 24]. Previous research has characterised the distinctions between wellbeing and mental illness. Keyes’s complete mental health model, for example, shows that wellbeing (defined as the presence of positive feelings and positive psychological and social functioning) and mental illness (defined as the presence of one or more of a range of DSM-III mental disorders) are separate but moderately correlated constructs (r ~ −0.5) [25]. In short, the evidence indicates that “the absence of mental illness does not imply the presence of *mental health* (as defined by Keyes, italics added by author), and the absence of *mental health* does not imply the presence of mental illness” [24].

Most research on worker wellbeing to date has focused specifically on work context or domain-specific wellbeing, most prominently job satisfaction [16, 17, 26, 27]. This has yielded valuable insights into a range of areas including work and organisational performance, employee engagement, employee retention/intentions-to-quit, organisational citizenship behaviours, and various aspects of productivity [15–17, 23, 26, 27]. Recent interest in worker wellbeing continues to be driven by relationships with performance and productivity, but also by the changing nature of workplace health and safety [16]. Many workplace injuries and occupational diseases have been controlled in industrialised countries, and current concerns about worker health are driven more by chronic disease, disability, and workability—which usually occur due to long periods of exposure to risk and protective factors which may include both work-related and other causes [15, 16]. This has led to a growing interest in broad and comprehensive measures of worker wellbeing to complement the previous emphasis on work domain-specific wellbeing measures such as job satisfaction [16, 19].

In this paper, we examine a global subjective wellbeing measure, which integrates satisfaction with various life domains, in a working population sample in comparison to a more commonly-used measure of ill-mental health. To complement previous research demonstrating the relationship between psychosocial working conditions and mental health problems, we present analyses relating psychosocial job quality to global subjective wellbeing, and include analyses relating psychosocial job quality to a measure of mental ill-health for direct comparison. This contributes to the need to better understand the determinants of worker wellbeing [16], including comparison of the similarities and differences in the relationships between work characteristics and the complementary domains of wellbeing and ill-health.

## Methods

### Design and sample

Ten to Men is a national cohort study of Australian males [28, 29]. Wave 1 data collection took place from October 2013 to July 2014, resulting in detailed information being provided by 15,988 males aged 10–55 living in close to 14,000 households. Wave 2 data collection has begun and was completed in June 2016. The response fraction at Wave 1 was 35 % of confirmed in-scope males.

The cohort was recruited via a stratified, multi-stage, cluster random sampling strategy that involved approaching eligible males residing in private dwellings, with separate cluster samples drawn from regional strata to ensure over sampling of males from regional areas. All private dwellings in sampled areas were enumerated and all males within the target age range in those dwellings were invited to participate. Interviewers collected household-level information including details of all males in the household regardless of whether they were participating or not. All participants provided informed written consent. Data were collected by personal interview for males aged 10–14, and self-complete paper hard copy questionnaire for males aged 15 years and older. The questionnaires covered a range of dimensions including social, demographic, health and economic conditions [29]. The analyses presented in this paper are restricted to males aged 18–55 at baseline as these participants would have had the opportunity to complete secondary education and to participate in the labour market. The Human Research Ethics Committee at the University of Melbourne approved the pilot studies and the main Wave 1 data collection.

### Outcome variables

We used two outcome measures for this analysis: The Personal Wellbeing Index – Adult (PWI-A) and the Mental Component Summary (MCS) of the Short Form 12 (SF-12). The PWI-A is a multi-item scale designed to measure subjective wellbeing that has been widely used to characterise and monitor population level wellbeing in Australia. It contains seven items about satisfaction, each one corresponding to a quality of life domain, and measured on an 11-point Likert scale (from 0, completely dissatisfied, to 10, completely satisfied): standard of living, health, achieving in life, relationships, safety, community-connectedness, and future security [30]. The seven items are summed to provide an overall score. These domains represent the deconstruction of satisfaction with ‘life as a whole’. Notably, satisfaction with job or work is not included, which makes the scale applicable to the full adult population including the unemployed and those not in the labour force due to caring responsibilities, disability, retirement, or other reasons. The range of the PWI-A is from 1–100, with 100 representing optimal overall life satisfaction. The seven domains load consistently on a single stable factor that account for 50 % of the variance in Australian samples and the Cronbach’s alpha lies between 0.70 and 0.85 [30]. The mean score on the PWI-A in Wave 1 of Ten to Men was 70.33 with a standard deviation of 17.23.

The MCS is a summary measure of mental health derived from the SF-12 health survey [31]. The SF-12 is a widely used measure of health status, has been validated for use in the Australian population [32]. The scores are standardised to a mean of 50 and an SD of 10 (range from 1–100), with 100 representing optimal functioning and mental health. The MCS mean score in the Ten to Men Wave 1 was 49.89, with a standard deviation of 9.23.

### Exposure variables

The psychosocial job characteristic items included in Ten to Men survey (control, demands and complexity, job insecurity, unfair pay and overall psychosocial job quality) were used to compute a multidimensional measure of psychosocial job quality. Previous publications document the full details of the construction and validation of the job quality measure [9, 11] as well as its use by our investigator group in other studies [12, 33]. In brief, factor analysis and structural equation modelling identified three separate factors, which were labelled: job demands and complexity (three items, alpha = 0.70, e.g.: “my job is complex and difficult”); job control (three items, alpha = 0.64, e.g.: “I have freedom to decide how I do work”); and perceived job security (three items, alpha = 0.82, e.g.: “I have a secure future in my job”). An additional single item assessing whether respondents considered that they were paid fairly for their efforts at work (“I get paid fairly for the things I do in my job”) was included as a fourth factor measuring one aspect of effort-reward imbalance. Each of these four individual factors was associated with more widely used measures of job demands and control, and other employment conditions such as casual status, hours worked and shift work [9].

To create the overall psychosocial job quality measure, each factor was dichotomized to identify the quartile experiencing the greatest adversity and the composite measure was constructed by summing the number of adverse psychosocial job conditions (high job demands and complexity, low job control, high job insecurity and unfair pay). Because of the small number of respondents reporting all four job adversities, this composite scale was top-coded at three, yielding categories of optimal jobs (no psychosocial adversities), and one, two, and three or more psychosocial adversities (poorest quality jobs).

### Other variables

Potential confounders of the job stressor—mental health/wellbeing relationships were selected on the basis of past literature. These included age (measured continuously, 18–55 years), occupational skill level (low [sales, machinery workers, and labourers], medium [technical and trade workers, community and personal service workers, and clerical and admin workers], and high [managers and professionals] according to the Australian and New Zealand Standard Classification of Occupations occupational groupings [34]), employment arrangements (permanent, casual or labour hire, fixed term or self-employed), working hours in main job (up to and including 40 h, over 40 h) [35] and presence of disability or long term health condition (yes or no) using the Washington Group Disability questions [36].

### Statistical analysis

Variables were summarised descriptively using frequencies, means, and standard deviations. We analysed data on all adult males with valid measures for the PWI-A, MCS, and psychosocial job quality (employed males, aged 18–55 years at baseline). We modeled the corsspsectional association between psychsocial job stressors and the PWI-A and MCS outcome separately using multiple linerar regression. Firstly, we modelled PWI-A (model 1) and MCS (model 2) as outcomes in relation to the four individual psychosocial job stressors as continuous variables (job control, job demands, job security and fairness of pay, mutually adjusting each for the others). Secondly we modelled the PWI-A (model 3) and MCS (model 4) outcomes in relation to overall psychosocial job quality (as an ordinal variable reflecting 1 adversity, 2 adversities, 3 or more adversities, considered relative to the omitted reference category of optimal job quality). We controlled for confounding by including a number of relevant covariates (age, occupational skill level, employment arrangements, working hours and presence of long term health condition or disability and education).

## Results

Table 1 presents the employment status of the full baseline sample aged 18–55. The majority of the sample was employed, with 8.4 % unemployed and looking for work and a smaller percentage not in the labour force (6.1 %). Employed respondents showed the highest average levels of mental health and subjective wellbeing.

**Table 1:** Employment status of Australian males aged 18 and above: Wave 1 of the Ten to Men cohort

	*N* (%)	SF-12 Mental Component Score Mean (SD)	Subjective Wellbeing Score Mean (SD)
Employment Status			
Employed	11,511 (85.6)	50.60 (8.581)	72.18 (15.780)
Unemployed & looking for work	1124 (8.4)	46.19 (11.251)	59.55 (19.776)
Neither working nor looking for work	821 (6.1)	44.68 (12.022)	58.99 (22.511)
Total		49.89 (9.234)	70.33 (17.231)
*N*	13,456	12,767	13,031

The eligible sample for describing working conditions included 11,511 employed men. The analytic sample for overall psychosocial job quality and mental health was 9,633 (83.6 % of eligible sample) and for subjective wellbeing was 9,777 (84.9 % of eligible sample). Table 2, presents demographic information on the eligible sample. The largest age group was those aged 50–55 years (34 %). Only a small number of these employed participants reported the presence of a disability or long term health condition (5.1 %). The majority of the sample had completed year 12, or high school/secondary education (61.4 %). Many had trade qualifications (27.5 %) or some university degree (29.5 %).

**Table 2:** Socio-demographics of Australian working males: Wave 1 of the Ten to Men cohort

Characteristics	Summary measure
Age (*N*, %)	
18-29 years	2498 (21.7)
30-39 years	3207 (27.9)
40-49 years	1895 (16.5)
50-55 years	3911 (34.0)
*N*	11,511
Disability (*N*, %)	
Working without disability	10,808 (94.9)
Working with disability	578 (5.1)
*N*	11,386
Completed year 12 (*N*, %)	
Did not complete year 12	4391 (38.64)
Did complete year 12	6974 (61.36)
*N*	11,365
Highest qualification after school (*N*, %)	
No other qualification	2575 (23.38)
Trade qualification	3248 (29.50)
Non university degree	2076 (18.85)
Some university degree	3033 (27.54)
Other	80 (0.73)
*N*	11,012

Table 3 presents descriptive data on employment and working conditions for working participants. The majority of these males were employed in permanent jobs (69.9 %), and the largest group was in a high occupational skill level (38.4 %). Almost one in 10 respondents were working in more than one job (9.8 %), and there was a high prevalence of long working hours (>40/week) when all jobs were included (49.9 %), and a similarly high level when asking about working hours in the respondents main job only (47.1 %). Nearly one in four respondents reported doing night shift work (23.4 %).

**Table 3:** Working conditions of Australian working males: Wave 1 of the Ten to Men cohort

Characteristics	Summary measure
Job control (Mean, SD)	3.39 (1.56)
Job demands (Mean, SD)	3.88 (1.31)
Job security (Mean, SD)	3.95 (1.36)
Fair pay (Mean, SD)	3.60 (1.70)
Overall job quality (*N*, %)	
Optimal	3966 (36.11)
1 adversity	4037 (36.76)
2 adversities	2239 (20.39)
3 or more adversities	740 (6.74)
*N*	10,982
Employment arrangement (*N*, %)	
Permanent	7904 (69.9)
Fixed term	1201 (10.6)
Casual/temporary	454 (4.0)
Self employed	1750 (15.5)
*N*	11,309
Occupational skill level (*N*, %)	
High	4201 (38.4)
Medium	3945 (36.1)
Low	2798 (25.6)
*N*	10,944
Working hours in all jobs (*N*, %)	
34 and under	1550 (14.7)
35 to 40	3644 (35.4)
41 to 48	1881 (18.3)
49 to 59	1791 (17.4)
60 h plus	1463 (14.2)
*N*	10,329
More than one job (*N*, %)	
No	8976 (90.2)
Yes	973 (9.8)
*N*	9949
Night shift work (*N*, %)	
No	8695 (76.6)
Yes	2652 (23.4)
*N*	11,347

### Regression models

Results from the adjusted linear regression models show associations between the four specific psychosocial job quality indicators (control, demand, security, fair pay and overall) and both the subjective wellbeing (Model 1) and mental health (Model 2) outcomes, including after adjustment for age, occupational skill level, employment arrangements, presence of long term health condition or disability, working hours and education (Table 4). Job security showed the strongest association with both outcomes, followed in descending order by job control, fairness of pay, and job demands.

**Table 4:** Subjective wellbeing and mental health: Multivariate regression models with individual psychosocial job quality indicators mutually adjusted, working males, Wave 1 of the Ten to Men cohort

	Model 1 Personal Wellbeing Index			Model 2 SF-12 Mental Health		
Individual stressors	Coef.	[95 % CI]		Coef.	[95 % CI]	
Job Control	1.25	1.04	1.47	0.54	0.42	0.67
Job Demand	0.25	<0.01	0.50	−0.30	−0.44	−0.15
Job Security	3.08	2.86	3.31	1.13	1.00	1.26
Fair Pay	1.25	1.07	1.42	0.38	0.27	0.48
Occupational Skill level						
High	*ref*			*ref*		
Medium	−1.35	−2.14	−0.56	0.41	−0.05	0.87
Low	−2.24	−3.15	−1.33	0.54	0.01	1.06
Employment arrangements						
Permanent	*ref*			*ref*		
Casual/temporary	1.02	−0.04	2.07	0.62	<0.01	1.23
Fixed term	0.53	−0.96	2.03	−0.10	−0.97	0.77
Self employed	0.46	−0.42	1.34	−0.81	−1.32	−0.30
Hours worked in main job						
up to 40 h	*ref*			*ref*		
over 40 h	0.99	0.39	1.59	0.11	−0.24	0.46
Disability						
No	*ref*			*ref*		
Yes	−9.29	−10.63	−7.95	−5.75	−6.54	−4.96
Completed year 12						
Not completed year 12	*ref*			*ref*		
Completed year 12	0.49	−0.21	1.18	−0.14	−0.55	0.26
Highest qualification after school						
No other qualification	*ref*			*ref*		
Trade qualification	2.13	1.28	2.98	0.67	0.176	1.17
Non university degree	0.17	−0.75	1.08	−0.39	−0.917	0.15
University degree	1.44	0.49	2.39	−0.16	−0.705	0.39
Other	−3.54	−7.01	−0.07	−0.81	−2.84	1.22

Table 5 shows the results for the overall measure of psychosocial job quality for both the subjective wellbeing (Model 3) and mental ill-health (Model 4) outcomes. There was a strong stepwise decrease in subjective wellbeing with increasing psychosocial job adversities. For men in the poorest quality jobs (with 3–4 adversities) there was a 13.00 (95 % CI −14.21 to −11.77) point decrease in average subjective wellbeing compared to men in optimal jobs. A similar pattern of results was seen for mental health as for subjective wellbeing. There was a step-wise decrease in mental health with increasing psychosocial job adversities. For those with 2 adversities, there was a 3.52 (95 % CI −3.99 to −3.05) decrease in mental health, and for those with three or more adversities the decrease was 5.64 points (95 % CI −6.34 to −4.93) compared to men working in optimal jobs. Although the magnitude of associations was smaller for the mental health outcome (coefficients of 4.1, 8.9, and 13.0 points lower on the wellbeing scale for exposure to one, two, or 3+ adverse stressors compared to the corresponding coefficients of 1.6, 3.5, and 5.6 for mental health), they are comparable effect sizes as proportions of the respective standard deviations of the two outcome measures, as can be gleaned by comparison of the SD estimates presented in Table 1.

**Table 5:** Subjective wellbeing and mental health: Multivariate regression models with overall psychosocial job quality indicator, working males, Wave 1 of the Ten to Men cohort

	Model 3 Personal Wellbeing Index			Model 4 SF-12 Mental Health		
Overall job quality	Coef.	[95 % CI]		Coef.	[95 % CI]	
Optimal				*ref*		
1 adversity	−4.14	−4.84	−3.45	−1.65	−2.04	−1.26
2 adversities	−8.90	−9.73	−8.06	−3.52	−3.99	−3.05
Poorest quality jobs	−13.00	−14.21	−11.77	−5.64	−6.34	−4.93
Occupational Skill level						
High	*ref*			ref		
Medium	−2.03	−2.84	−1.22	0.29	−0.17	0.75
Low	−3.46	−4.36	−2.56	0.42	−0.09	0.93
Employment arrangements						
Permanent	*ref*			ref		
Casual/temporary	−0.59	−1.67	0.48	0.27	−0.34	0.876
Fixed term	−0.82	−2.36	0.73	−0.62	−1.50	0.25
Self employed	0.61	−0.25	1.46	−0.67	−1.15	−0.19
Hours worked in main job						
up to 40 h	*ref*			ref		
over 40 h	1.59	0.98	2.21	0.20	−0.15	0.55
Disability						
No	*ref*			ref		
Yes	−9.84	−11.23	−8.44	−5.97	−6.77	−5.17
Completed year 12						
Not completed year 12	*ref*			ref		
Completed year 12	0.44	−0.29	1.17	−0.17	−0.58	0.24
Highest qualification after school						
No other qualification	*ref*			ref		
Trade qualification	2.17	1.28	3.05	0.58	0.08	1.08
Non university degree	0.19	−0.76	1.15	−0.48	−1.02	0.06
University degree	1.56	0.58	2.54	−0.26	−0.81	0.30
Other	−3.00	−6.61	0.62	−0.80	−2.85	1.25

## Discussion

Our results suggest that psychosocial job quality is related in a step-wise or dose–response fashion to decreasing general life satisfaction. This extends previous knowledge in that most previous research has related job stressors and other work characteristics to work-specific wellbeing measures such as job satisfaction. The observed dose–response relationship was qualitatively similar to the results from the analysis included for direct comparison purposes: psychosocial job quality and mental health. While it has been previously established that mental health, and health in general, is a determinant of global and work-specific wellbeing, less is known about how similar or different their relationships with job stressors might be [15, 16, 37].

The mental health outcome analysis was also included for validation purposes. The psychosocial job quality index was developed and has been tested in relation to health outcomes in only one cohort study thus far: the Household Income and Labour Dynamics in Australia (HILDA) study [10, 11]. Our Ten to Men results are consistent with HILDA results which showed a dose–response relationship between the psychosocial job quality index and mental health [11, 33] (as well as physical health [11] and sickness absence [12]).

There is growing policy and practice interest in worker wellbeing, as distinct from worker ill-health. This includes the possibility of including wellbeing measures in worker health policy and conducting quantitative risk assessments of wellbeing in relation to various determinants in similar way, for example, to risk assessments conducted for occupational cancer risks in relation to asbestos or benzene exposures [16]. Our results suggest that for this measure of wellbeing, quantitatively assessing wellbeing levels in relation to combined exposures to adverse working conditions is feasible, should such results be replicated for psychosocial and other occupational exposures in prospective studies. The differences observed across the range of psychosocial job quality observed were on the order of ½ standard deviation for each of the two outcome measures, which is generally acknowledged as a minimum clinically important difference [38]. The effect sizes for each outcome, in more concrete terms, were on a par with the presence of a disability or long-term health condition (Table 5). Further, the sample mean PWI score of 70.33 is lower than the Australian normative range for males of between 73.0 and 76.5 [39]; a 13 point lower mean PWI for those reporting the worst psychosocial job quality puts those respondents below 60 on the PWI scale, indicating a high risk of mental health problems.

The study of psychosocial working conditions in relation to wellbeing, as a complement to health outcomes, warrants further study. The qualitatively similar associations between psychosocial job quality and the two outcomes suggests that the PWI and SF-12 mental health overlap as constructs to some extent, as has been shown for other mental wellbeing and illness outcomes (as outlined in the Introduction section). However, there were notable differences in association patterns for some covariates. For example, occupational skill level differed in both direction and significance in relation to PWI and SF-12 mental health, with a strong step-wise negative association between PWI and decreasing skill level versus a non-significant positive association with SF-12 mental health. The relationships between psychosocial job quality and positive mental health/wellbeing and ill-mental health also require longitudinal study for validation of the results presented here as well as further understanding of the relationships between the wellbeing and illness outcomes. A recent longitudinal analysis, for example, showed that positive mental health buffered the adverse impacts of job stress on ill-mental health [40]. Future research will need to consider the potential for complex interactions between psychosocial job stressors, wellbeing and illness outcomes.

The results presented in this paper are also limited in various ways. Most importantly, our analyses are limited by their cross-sectional nature and by residual confounding. Further, while Ten to Men is a national sample, it is not representative of the full male working population, thus limiting generalisability. The overall estimated response fraction of 35 %, while comparable to other more recently established health cohort studies, limits generalisability [28, 29]. While there is a similar proportion of indigenous Australians in the Ten to Men cohort as the comparable population recorded in the 2011 Australian Census, Ten to Men participants are also older, and more likely to be Australian born compared to the 2011 census [28, 29]. Further, higher proportions reside in inner and outer regional areas, reflecting the fact that Ten to Men deliberately oversampled in these areas [28, 29]. Further, males from culturally and linguistically diverse backgrounds were functionally excluded as study materials could not be produced in languages other than English due to resource constraints. These various limitations manifest in the over-representation of older, higher skill-level, permanently-employed working men, and the under-representation of younger, lower skill level, and casual or temporarily-employed workers. This likely results in higher psychosocial job quality in the Ten to Men sample compared to the working population compared to other cohorts such as the Household Income and Labour Dynamics in Australia (HILDA) cohort and thus underestimation of the psychosocial job quality—mental health/wellbeing associations observed [33, 41]. Despite the limitations of the design and sample, including the potential for common method variance or dependent misclassification bias to artefactually inflate associations, the observed associations in this study for the mental health outcome are complemented by longitudinal and fixed effects within-person analyses that also show associations between psychosocial job quality and mental health [11, 33, 42]. We are not aware of such analyses as yet having been conducted for global wellbeing outcomes.

### Future study of work, health and wellbeing in the Ten to Men cohort

Ten to Men represents a valuable new resource for the longitudinal and life course study of work and wellbeing, as well as work and adverse health outcomes in male populations. The collection of detailed data on a wide range of social as well as other determinants of health will enable study of the intersection of work with other major influences on male health and wellbeing. The second wave of data collection is anticipated to conclude in 2016. Ten to Men data are available to researchers via a request and review process [28].

## Conclusions

The results of this analysis extend the study of psychosocial working conditions to global subjective wellbeing as an outcome. A dose–response relationship was observed with substantially declining wellbeing with increasing number of job stressor exposures. A qualitatively similar relationship was observed for a scaled mental health outcome. These results, though limited by their cross-sectional nature and requiring further study, add further justification to the case for intervening to reduce exposure to job stressors, as this may prevent the deterioration of wellbeing as well as preventing mental health problems and common mental disorders.

## Abbreviations

ABS: Australian Bureau of Statistics, ANZSCO, Australia/New Zealand Standard Classification of Occupation
HILDA: Household Income & Labour Dynamics in Australia (panel study)
MCS: Mental Component Score of the Short Form-12
PWI-A: Personal Wellbeing Index—Adult
